# Genomic profiling of enterotoxigenic Escherichia coli toxins and adhesins in livestock isolates from Kenya

**DOI:** 10.1099/mgen.0.001515

**Published:** 2025-09-25

**Authors:** Samuel M. Njoroge, Benard W. Kulohoma, Laura C. Falzon, Timothy K. Kamanu, Kelvin Momanyi, Patrick Muinde, Maurice K. Murungi, Allan Ogendo, Joseph Ogola, Lilian Wambua, Erastus Kangethe, Jonathan Rushton, Mark Woolhouse, Nicholas R. Thomson, Samuel Kariuki, Astrid von Mentzer, Eric M. Fèvre

**Affiliations:** 1 Department of Biochemistry, Centre for Biotechnology and Bioinformatics, University of Nairobi, Nairobi, Kenya; 2 International Livestock Research Institute, Nairobi, Kenya; 3 Centre for Microbiology Research, Kenya Medical Research Institute, Nairobi, Kenya; 4 IAVI, Nairobi, Kenya; 5 Institute of Infection, Veterinary and Ecological Sciences, University of Liverpool, Liverpool, UK; 6 School of Mathematics, CBPS College, University of Nairobi, Nairobi, Kenya; 7 World Animal Protection, Nairobi, Kenya; 8 Veterinary Department, Busia County Government, Busia, Kenya; 9 Veterinary Department, Bungoma County Government, Bungoma, Kenya; 10 Faculty of Veterinary Medicine, University of Nairobi, Nairobi, Kenya; 11 Centre for Immunity, Infection and Evolution, University of Edinburgh, Scotland, UK; 12 Usher Institute of Population Health Sciences & Informatics, University of Edinburgh, Scotland, UK; 13 Wellcome Sanger Institute, Hinxton, UK; 14 London School of Hygiene and Tropical Medicine, Keppel Street, London, UK; 15 Kenya Medical Research Institute, Nairobi, Kenya; 16 Department of Microbiology and Immunology, Institute of Biomedicine, Sahlgrenska Academy, University of Gothenburg, Gothenburg, Sweden; 17 SciLifeLab, University of Gothenburg, Gothenburg, Sweden

**Keywords:** A_2_B_5_ toxins, adhesins, cytolethal-distending toxin, enterotoxigenic *Escherichia coli*, Kenyan livestock, pertussis-like toxins, Shiga toxin-producing *Escherichia coli*, reversed order AB_5_ toxin

## Abstract

Enterotoxigenic *Escherichia coli* (ETEC) is a significant cause of diarrhoea in livestock and humans. The epidemiology of ETEC in animals remains understudied, prompting an investigation into the virulence factors and associated adhesins of ETEC in livestock from Western Kenya. Also, there is limited evidence supporting the role of livestock as possible zoonotic reservoirs for ETEC. ETEC strains harbour colonization factors/adhesins and enterotoxins, with animal ETECs exhibiting various adhesins (F4, F5, F6, F17, F18 and F41). Enterotoxins include heat-labile (LT) and heat-stable (ST) toxins and are further divided into LT-I and LT-II and STa and STb, respectively. Additional toxin combinations occur, with ETEC and Shiga toxin-producing *E. coli* (STEC) hybrids garnering public health significance. Here, we analysed faecal and mesenteric lymph node samples from diverse livestock across three Western Kenyan counties (Busia, Bungoma and Kakamega), using whole-genome sequencing. *In silico* screening determined the presence of AB_5_ and A_2_B_5_-like toxin genes, including cytolethal distending toxin (*cdt*ABC) along with associated adhesins. To broaden the screening panel, adhesin genes identified were further characterized to identify both known and novel alleles, particularly focusing on human-ETEC colonization factors. Two *estA* alleles (*estA-*4-06, *estA-*6-02) and six *eltAB*-II toxin alleles (*eltAB*-II-a2-01, *eltAB*-II-a3-01, *eltAB*-II-c1-02, *eltAB*-II-c6-03, *eltAB*-II-c6-04 and *eltAB*-II-c7-02) were identified in livestock. Hybrid ETECs identified were ETEC/STEC present in 6.7% (4/60) of ETEC strains and ETEC with *cdtABC* type I. An A_2_B_5_-like tripartite toxin, potentially resembling the typhoid toxin, was detected in 8.7% (4/46) of the *eltAB*-II-positive strains. It may have unique effects on enterocytes distinct from known toxins. These findings expand our understanding of ETEC pathogenicity and genetic diversity in animal reservoirs, while also highlighting potential zoonotic risks. They broaden the toxin repertoire, offer adhesin-based vaccine candidates for livestock and provide valuable insights for future vaccine development and public health strategies in the Lake Victoria Crescent ecosystem and beyond.

**Impact Statement**

Enterotoxigenic *Escherichia coli* (ETEC), a significant cause of diarrhoea in livestock, is attributable to economic losses. By analysing ETEC livestock samples in Western Kenya, our study unveiled toxin repertoire, putative adhesins, phylogroups, multilocus sequence types, plasmid replicons and serotypes.

This study provides new insights on AB_5_ and A_2_B_5_ toxins and putative adhesins, as well as their associated alleles in ETEC in livestock within the broader Lake Victoria Crescent ecosystem of eastern Africa.

## Data Summary

Short reads and assemblies generated in this study are available at the NCBI under BioProject PRJEB33365. Primer sequences, multilocus sequence typing allele profiles for new entries, sequence run accession numbers, metadata for each genome and specific accession numbers for toxin alleles are found in the Supplementary Material tables. Isolates in this study are stored in the Azizi Biorepository and are available on official request.

## Introduction

Enterotoxigenic *Escherichia coli* (ETEC) is a significant cause of diarrhoea in young farm animals [1]. ETEC pathogenicity involves the presence of fimbrial/non-fimbrial adhesins and the production of heat-stable (ST) and/or heat-labile (LT) enterotoxins. The initial step of ETEC pathogenesis entails its attachment to the intestinal epithelium through fimbrial or non-fimbrial adhesins, also called colonization factors (CFs) antigens, by recognizing host-specific receptors on enterocytes, leading to subsequent colonization [2].

Among the enterotoxins produced by ETEC, heat-labile toxins belong to the ADP-ribosylating toxin (ART) family and have an AB_5_ structure. They consist of an enzymatically active subunit A and a pentameric subunit B, which binds to gangliosides and galactose-containing surface receptors on enterocytes [3]. There are two subfamilies of heat-labile toxins: LT-I (*eltAB*-I) and LT-II (*eltAB*-II). LT-I shares ~77% nt sequence identity and has a comparable 3D structure of the subunit B to cholera toxin (CT), while LT-II exhibits limited sequence identity (<14%) to CT and LT-I [4]. LT-I-producing ETEC strains are primarily associated with diarrhoea in humans and piglets [5,6]. LT-II is further divided into three subtypes: LT-II-a, LT-II-b and LT-II-c with 1, 2 and 9 alleles, respectively [7]. LT-II-a and LT-II-b-producing ETEC isolates are associated with diarrhoea in pigs, buffalo and cattle, while their occurrence in humans is rare [8]. The recently described LT-II-c subtype was initially isolated from diarrhoeic stool samples from dead ostriches and is less cytotoxic compared to CT, LT-II-a and LT-II-b [9,10].

In the past two decades, most of ETEC research has focused on LT-I toxins. The molecular mechanism of action of LT-II toxins (LT-II-a and LT-II-b) remains unclear, as they do not induce fluid accumulation in the adult rabbit ileum and do not bind to ganglioside monosialotetrahexosylganglioside. Additionally, these toxin subtypes are chromosomally encoded and their absence in conjugative plasmids has limited their studies [11].

Heat-stable toxins, on the other hand, are divided into two main groups, STa [*estA,* split into STa-p (*estA*-1,4-6) and STa-h (*estA*-2 and 3)] and STb (*estB*), based on their initial host isolation (porcine or human), and they do not share aa or tertiary structure similarity [7,12]. Currently, 15 porcine *estA(p*) and 4 human *estA(h*) alleles have been identified, while there are only 4 known alleles for *estB* encoding STb [7,13]. STa induces secretory diarrhoea by binding to a guanylate cyclase C receptor, which leads to elevated levels of cyclic GMP (cGMP) and activates the cystic fibrosis transmembrane conductance regulator through cGMP-dependent protein kinase II phosphorylation. The outcome is the secretion of Cl^−^ and H_2_O and the inhibition of Na^+^ absorption in the ileum [14]. In contrast, the cascade of events that lead to diarrhoea by STb remains unclear and biologically there are differences; for instance, STb does not significantly contribute to diarrhoea caused by ETEC in neonatal pigs [15].

In addition to the aforementioned toxins, genes encoding other AB_5_ toxins have been identified in ETEC strains and should be included in the toxin profile screen. These include, but are not limited to, Shiga toxins (*stxAB*-1/2) and two pertussis-/subtilase-like toxin genes (*eplBA* and *ealAB*). Shiga toxin-producing *E. coli* (STEC) are characterized by the presence of two types of Shiga toxins encoded by *stxAB*-1 and *stxAB*-2 genes, with 4 Stx-1 subtypes (*stxAB-1a*, *stxAB*-1c, *stxAB*-1d and *stxAB*-1e) and 14 Stx-2 subtypes (*stxAB-2a-m* and *stxAB-2o*) reported to date [16]. Hybrid ETEC/STEC strains have been globally documented in diverse samples, including environmental, animal and human clinical samples, highlighting their potential as food-borne pathogens [17]. Two recently identified prophage-encoded pertussis-/subtilase-like toxins carried by LT-II-positive strains are EplBA and EalAB, which bind to sialic acid-containing glycoprotein ligands [18]. EplBA is closely related to pertussis-like toxin subunit B and A (PltBA), while EalAB exhibits codon sequence similarity to ArtAB, both of which are found in *Salmonella* spp., thereby expanding the ADP-ribosylating family of toxins [18]. Allele *ealAB*-6-01 is closely related to *artAB* than to the other alleles. Another member of the AB-toxin family is the A_2_B_5_ toxin type, represented by the typhoid toxin (TT) in *Salmonella* spp. and the cytolethal distending toxin (CDT) [19]. CDT is found in *E. coli* as well as other genera, including *Campylobacter*, *Shigella*, *Haemophilus*, *Actinobacillus*, *Helicobacter*, *Providencia* and *Pasteurella* [20,21]. In *E. coli*, CDT is produced by enteropathogenic *E. coli* (EPEC), enterohaemorrhagic *E. coli* (EHEC) and ETEC pathotypes [22]. A_2_B_5_ toxins have two enzymatic A-subunits and one binding pentameric B-subunit between the A-subunits. Two alleles of the TT are present in *Salmonella enterica* subsp. *enterica* serovar Typhi and *S. enterica* subsp. *enterica* serovar Javiana, sharing 99% sequence similarity [23,24].

In addition to enterotoxins, ETEC from animals, particularly pigs and cattle, are known to possess well-characterized adhesins such as F4 (K88), F5 (K99), F6 (987P), F17, F18 and F41 fimbriae [25]. The primary animal host for ETEC exhibiting F4 (K88) fimbriae is pigs, with significant economic losses linked to the death of neonatal and weaned piglets [26]. The F4 operon consists of ten genes (*faeA-J*). Genes *faeA* and *faeB* function as regulators, while *faeD* acts as the transport protein/usher and *faeE* as the periplasmic chaperone. Genes *faeC*, *faeF* and *faeH* encode minor subunits contributing to the fimbrial structure, and *faeG* serves as the major pilin subunit, whereas the roles of *faeI* and *faeJ* remain unclear, particularly in relation to expression [27]. Among the ten genes, *faeG* and *faeI* exhibit the most variation, with 19 and 17 alleles identified, respectively. The *faeG* gene is further categorized into four clusters, *faeG-ab*, *faeG-ac*, *faeG-ad* and *faeG-W*, comprising 3, 14 and 1 allele(s) each, respectively [7].

The CS31A adhesin, encoded by the *clp* operon, closely resembles the *fae* operon, which encodes the F4 fimbriae, in its gene organization, and adhesion to the human colon carcinoma cell line Caco-2 and porcine neonatal jejunal epithelial cell line IPEC-J2 [28,30]. However, structural differences are observed, as CS31A appears as a capsule surrounding the bacterial surface under an electron microscope, in contrast to F4, which presents as fimbrial appendages and adheres to porcine or bovine intestinal epithelial cells [31].

F5 and F41 fimbriae are observed in ETEC strains isolated from diarrhoeic young ruminants [32]. ETEC from diarrhoeic neonatal piglets is associated with F6 fimbriae, whereas diarrhoeic weaned piglets are commonly linked to F18 fimbriae [33,34]

ETEC harbouring F17 adhesin has been detected in the faeces of diarrhoeal or septicaemic lambs and calves [35,36]. F17 fimbriae operon has four genes known as *f17A* (major fimbrial subunit), *f17C* (transport protein/usher), *f17D* (chaperone) and *f17G* (minor adhesive subunit). Variants are categorized mainly using the nt sequences of the major and minor fimbrial subunits (*f17A* and *f17G*). There are 17 alleles of *f17A* and 15 alleles of *f17G* currently available at the ETECFinder database [7]. However, little is known about the role of these adhesins in the pathogenicity of ETEC apart from the association with virulence.

Here, we analysed an ETEC collection isolated from cattle, goats, sheep and pigs in Western Kenya between the years 2017 and 2019, within a previously described integrated surveillance framework conducted under a mixed smallholder livestock production system [37]. The primary objective was to identify new or existing variants of enterotoxins in these livestock, along with any previously described or novel fimbrial-like adhesins. Specifically, the study aimed to (i) identify descriptive risk factors associated with ETEC colonization in livestock populations; (ii) detect and characterize allelic variants of enterotoxin genes; (iii) identify fimbrial adhesins, particularly those homologous to human CFs, to assess cross-species similarities; (iv) investigate the presence of hybrid ETEC strains, including ETEC/STEC and strains harbouring additional toxin genes such as CdtABC, pertussis-like toxins and A_2_B_5_-like toxins; and (v) provide genome-based insights into the zoonotic potential of livestock-associated ETEC strains and their implications for public health in the region.

By targeting an understudied region and multiple livestock types (cattle, goats, sheep and pigs), this study contributes to the pan-genome analysis of animal ETEC strains, offering insights into toxin and pathotype combinations with associated adhesins resembling human CFs, possibly indicating cross-species adaptation. The findings underscore the plasticity of *E. coli* genomes, their capacity to acquire diverse virulence factors (VFs) and the role of animals as potential reservoirs, emphasizing the importance of comprehensive global surveillance for understanding ETEC diversity. Subsequently, new toxin alleles will add to the VirulenceFinder database at the Center for Genomic Epidemiology and other public databases.

## Methods

### Sampling, culture conditions and strain collections

A total of 1,052 *E. coli* isolates derived from faecal samples of apparently healthy or asymptomatic livestock were screened for ETEC in this study. The faecal samples included cattle (*n*=582), goats (*n*=241), pigs (*n*=121) and sheep (*n*=108). These isolates were conveniently selected from an integrated surveillance programme for zoonotic diseases conducted between March 2017 and May 2019. The sampling was conducted in selected livestock markets and slaughterhouses/slabs in the Busia, Bungoma and Kakamega counties of Western Kenya.

During the 2-year period, each sentinel site was visited every 4 weeks, and up to ten animals at each site were sampled. The detailed sampling procedures have been described previously [37]. Faecal consistency was categorized as diarrhoeic, haemorrhagic, hard, mucoid or normal.

In addition to the faecal samples, *E. coli* isolates from 41 aseptically collected mesenteric lymph nodes (MLNs) of slaughtered pigs were included in the study for exploratory purposes, without adhering to predefined selection criteria. This summed the total number of *E. coli* isolates from pigs tested for ETEC to 162.

Initial culturing of the samples was done in the Busia field laboratory, and isolates were profiled as *E. coli* biochemically at the International Livestock Research Institute (ILRI) Nairobi campus.

Initially, a pea-sized stool sample/stomached MLN was emulsified in 3 ml buffered peptone water and incubated overnight at 37 °C. Subsequent plating on McConkey agar to yield pure distinct lactose-positive colonies was done at 37 °C for 18–24 h. The colonies were re-plated in nutrient agar. Three pure lactose-positive colonies were cryo-preserved separately in tryptone soy broth with 15% glycerol. A fourth vial containing random, mixed same-animal colonies from the primary culture was used for initial toxin screening by conventional PCR; if positive, pure colonies from any of the remaining three vials were selected for further analysis. All media used were from Oxoid (Basingstoke, UK).

### PCR-based screening of toxins

A simplex PCR assay was performed for the initial screening of toxin genes (STh, STp, STb, LT-I and LT-II). Primers used for the uniplex conventional PCR assays targeting these toxins are listed in Table S1 (available in the online Supplementary Material). Isolates flagged as positive were subjected to DNA extraction.

### Descriptive statistics and risk factor analysis

Data preparation included complete-case analysis, excluding records with missing values for key variables (age, gender, livestock type, temperature, faecal consistency, premise and ETEC status). ETEC isolation was converted to a binary outcome (1=yes and 0=no). Age was categorized into three developmental groups (<1 year, 1–3 years, and ≥3 years), respectively, representing young, juvenile and adult. Rectal temperature status (normal/elevated) was determined using livestock-type-species-specific thresholds for bovine, ovine, caprine and porcine. Rectal temperature reference intervals were obtained from the international Merck veterinary manual [38]. Descriptive statistics were generated using stratified tables, presenting frequencies by ETEC status and livestock type, with between-group comparisons using chi-square tests. Results were formatted using TABLE1 and FLEXTABLE packages in (v4.2.0) (https://www.R-project.org/), with significance denoted at *α*<0.05.

### Genomic DNA isolation

ETEC isolates were incubated in 10 ml of Luria–Bertani broth (Mast Diagnostica GmbH, Reinfeld, Germany) overnight at 37 °C in a shaker (250 r.p.m.). The bacterial pellets were recovered after 10 min of centrifugation at 3,000 ***g***, and genomic DNA was extracted using the Wizard Genomic DNA Purification Kit (Promega, USA) in accordance with the manufacturer’s specifications. The purified DNA was suspended in 100 µl of Tris-EDTA buffer, stored at −20 °C and sent to the Wellcome Sanger Institute for sequencing. DNA concentrations were determined using the Qubit dsDNA BR Assay Kit (Invitrogen, Carlsbad, CA), and 50 ng of DNA was used for library preparations.

Libraries were prepared using the MGI FS Library Prep Set v2.1 (MGI Tech), following the manufacturer’s recommendations on fragmentation, adapter tagging, tagmentation and fragment standardization.

### Whole-genome sequencing, assembly and quality control

Next-generation sequencing for whole genome on pooled libraries was done on NovaSeq 6000 benchtop sequencer (Illumina) using the NovaSeq 6000 Reagent kit v1.5 (Illumina) for 300 cycles generating reads 150 bases long. Raw data reads were quality trimmed using CutAdapt v4.1 [39], keeping reads of at least 40 bp with a quality score of Q30.

Multiple de novo assembled reads were created using VelvetOptimiser v2.2 and Velvet v1.2.10 5 [40]. An assembly improvement step was performed on the ideal N50 assembly, and contigs were scaffolded using SSPACE v2.0 [41]. Genomes exceeding 6 Mb, or with more than 600 contigs, were excluded from further analysis. Annotation was performed with Prokka v1.5 [42] with customized ETEC toxin and adhesin genes, and contamination was assessed using Kraken 2 v1.1 pipeline [43].

### Identification of plasmids, sequence types, virulence, insertion sequences and phylogroups

ABRicate pipeline incorporating multiple databases was used for virulence screening relying on the Ecoli_VF, plasmid replicon identification based on the PlasmidFinder [44] and in silico serotyping using the EcOH [45]. Additionally, phylogroups were determined using ClermonTyper v1.0.0 [46] categorizing them into eight main phylogroups designated as A, B1, B2, C, D, E, F and clade I. Multilocus sequence typing (MLST) was done using MLST v2.19 (https://github.com/tseemann/mlst), PubMLST database (https://pubmlst.org) using the Achtman scheme [47,48]. New sequence types (STs) and clade complexes were identified and verified by submission of assembled contigs to pubMLST (https://pubmlst.org/organisms/escherichia-spp). Insertion sequences were screened using the ISEScan script available at https://github.com/xiezhq/ISEScan [49]. Three new MLST profiles are shown in Table S2.

### Phylogenetic analysis

Fifty-four ETEC genomes (90%, 54/60) with <600 contigs and size <6 Mb were selected. With Prokka-derived genome annotation files as inputs, Panaroo (v1.2.3) was used to define the core genome [50]. The parameters were set to ‘--clean-mode moderate --a core --core_threshold 0.99 t 12 --search_radius 10 --refind_prop_match 100’. SNP sites were used to extract SNP sites and constant sites for each ATGC nt. The inferred maximum-likelihood core tree phylogeny was generated with IQTree (v1.6.12) using the GTFR+F+I model with 1,000 fast bootstraps, incorporating SNP sites for branch length linear scaling [51,52]. Tree visualization with appended metadata was done using R (v4.2.0) (https://www.r-project.org/) with CRAN packages: GGPLOT2, GGTREE, PHYTOOLS, PHANGORN, GGNEWSCALE and COWPLOT loaded (CRAN: Available Packages By Name (r-project.org)).

### Genomic analysis of VFs

tblastx screening was performed with NCBI-blast v2.12.0 to identify adhesin and toxin operons with queries adapted from https://github.com/avonm/etec_vir_abricate. aa sequences for each adhesin and toxin gene in the locus were concatenated excluding intergenic regions, gene overlaps were corrected and the stop codons were removed for each variant and aligned MAFFT v7.475 preferably using the fasta-format, --globalpair, --maxiterate 16 and --inputorder as parameters, and the output aa alignment was used to obtain sequence identity percentages using an online tool available http://imed.med.ucm.es/Tools/sias.html. Signal peptide prediction was done using signal-6.0 available at https://github.com/fteufel/signalp-6.0 [53]. Locus gene-to-gene comparison was done using clinker v0.0.28 available at https://github.com/gamcil/clinker [54]. Hypervariable region containing aspartate-valine-serine-serine (DVSS) repeats was removed from *eplB* gene to obtain a non-biassed allele grouping.

## Results

### Sample types and initial screening with conventional PCR

A total of 1,052 *E. coli* isolates were selected from livestock faecal samples, including cattle (*n*=582), goats (*n*=241), pigs (*n*=121) and sheep (*n*=108). These *E. coli* isolates were a subset of isolates from livestock sampled in the parent ZooLink study, and that subset represented 26.7% (582/2,178), 40.4% (241/596), 26% (108/416) and 40.9% (121/296) of the sampled cattle, goats, sheep and pigs included in the broader surveillance [37]. Additionally, we also included 41 (13.9%, 41/296) MLN *E. coli* isolates from slaughtered pigs for exploratory purposes. In total, 1,093 *E. coli* isolates were tested using conventional PCR to determine the presence of ETEC toxins (LT-I, LT-II, STa and STb). The distribution of the 1,093 isolates by age, sex, rectal temperature, livestock type, premise, sample type and faecal consistency is detailed in Table 1.

**Table 1.** Summary statistics of samples tested for the presence of ETEC

Variable	ETEC isolated No (*N*=1033) (%)	ETEC isolated Yes (*N*=60) (%)	*P*-value
Livestock type			
Cattle	547 (53.0)	35 (58.3)	0.38
Goat	227 (22.0)	14 (23.3)	
Pig	153 (14.8)	9 (15.0)	
Sheep	106 (10.3)	2 (3.3)	
Age group			
Young<1	136 (13.2)	7 (11.7)	0.7
Juvenile 1–3	476 (46.1)	31 (51.7)	
Adult≥3	421 (40.8)	22 (36.7)	
Faeces consistency			
Normal	753 (72.9)	48 (80.0)	0.8
Diarrhoeic	177 (17.1)	7 (11.7)	
Haemorrhagic	2 (0.2)	0 (0)	
Hard	60 (5.8)	3 (5.0)	
Mucoid	41 (4.0)	2 (3.3)	
Rectal temperature			
Normal	966 (93.5)	54 (90.0)	0.43
Elevated	67 (6.5)	6 (10.0)	
Premise			
Livestock market	550 (53.2)	31 (51.7)	0.92
Slaughterhouse	483 (46.8)	29 (48.3)	
Gender			
Female	597 (57.8)	31 (51.7)	0.42
Male	436 (42.2)	29 (48.3)	
Sample type			
Faecal	994 (96.2)	58 (96.7)	1
MLN	39 (3.8)	2 (3.3)	

Summary statistics of samples tested for the presence of ETEC.

Out of the 1,093 samples tested, 99 (9.1%, 99/1,093) isolates tested positive for LT-I, LT-II, STa and STb by conventional PCR. One isolate was excluded due to a conflicting sample type from the parent metadata, resulting in a final dataset of *n*=98. From the final dataset, 38 (38.7%, 38/98) were subsequently found to be toxin negative by WGS. Conversely, 60 isolates were positive by both conventional PCR and whole-genome sequencing (WGS).

### ETEC distribution and risk factors

Among livestock, ETEC isolation proportion was 6.0% in cattle, 5.8% in goats, 5.6% in pigs and 1.9% in sheep. Notably, 12.2% of pig MLN samples were positive for ETEC compared with 3.3% of pig faecal samples, underscoring their value in surveillance for enteric pathogens. None of the evaluated risk factors, including age, faecal consistency, rectal temperature, sampling premise, gender or specimen type, were significantly associated with ETEC positivity. Statistical significance was set at *α*=0.05 (two-tailed), and exact *P*-values are shown in Table 1. These results suggest a broad and potentially physiological distribution of ETEC within the livestock population, with no clear demographic or physiological indicators of carriage.

### Whole-genome characteristics of animal ETEC strains

**Fig. 1.**
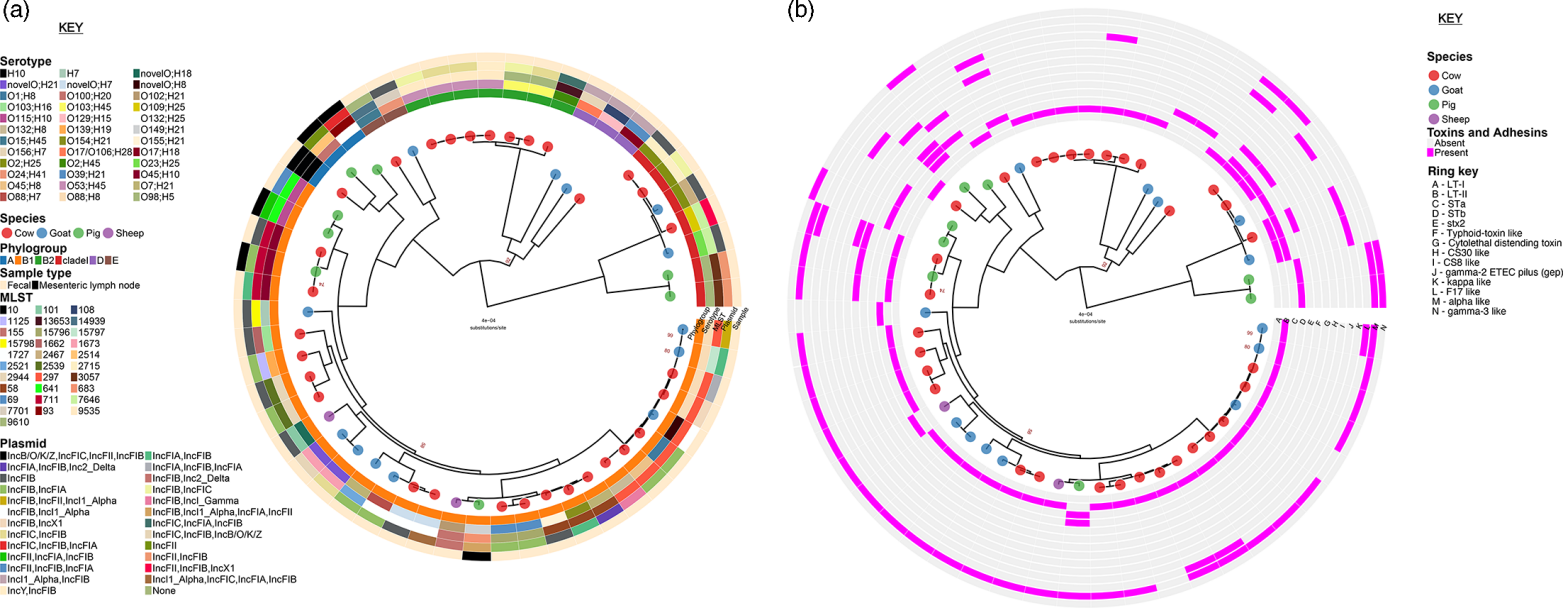
Serotypes, phylogroups, MLST, replicon plasmid profiles and sample types in relation to species are shown in (a) with a reference key on the left, while (b) illustrates associated toxins (rings A–G) and adhesins on rings H–G. Magenta colour denotes presence while the off-white colour denotes absence. The phylogenetic tree shown inside the rings is derived from the core genomes of 54 ETEC isolates from Western Kenya livestock.

Sixty animal ETEC isolates showed diversity across livestock types, MLST, phylogenetic groups and serotypes. MLST analysis identified 31 distinct STs (shown in Fig. 1, Table 2). Prevalent phylogroups were B1 (53.3%, 32/60), B2 (11.7%, 7/60), cryptic clade I (11.7%, 7/60), D (5%, 3/60), A (5%, 3/60) and E (3.3%, 2/60). Phylogroups C and F, which have been previously reported, were absent in this animal ETEC collection (Tables 2). Among these isolates, three new STs were identified, ST15796 (from cow) and ST15797-98 (from goats), all belonging to phylogroup B1, now included in the MLST Finder database.

**Table 2.** Genotypic features of livestock ETEC based on 54 isolates

Phylogroup (*n*)	MLST (*n*)	Serotype (*n*)	ETEC toxin (*n*)	Species (*n*)
A (3)	10 (1)	OX13:H10 (1)	LT-I (1)	Cow (1)
93 (1)	O132:H25 (1)	STb+CDT (1)	Pig (1)
2,514 (1)	O100:H20 (1)	STa+STb+stx (1)	Pig (1)
B1 (32)	58 (3)	O7:H21 (1), O155:H21 (1), O154:H21 (1)	LT-II (3)	Cow (3)
101 (1)	novelO:H18 (1)	STa (1)	Sheep (1)
155 (1)	O102:H21 (1)	LT-II (1)	Sheep (1)
297 (7)	O88:H8 (3), O45:H8 (1), O132:H8 (1), O1:H8 (1), novelO:H8 (1)	LT-II (7)	Cow (5), goat (2)
641 (2)	O115:H10 (1)	STb (1)	Pig (2)
683 (1)	O149:H21 (1)	STb+STa (1)	Pig (1)
711 (3)	O45:H10 (3)	LT-II+new A_2_B_5_ (1), LT-II+CDT+new A_2_B_5_ (2)	Cow (2), pig (1)
1,125 (1)	O139:H19 (1)	LT-II (1)	Cow (1)
1,662 (1)	O103:H16 (1)	LT-II (1)	Cow (1)
1,673 (2)	novelO:H21 (2)	LT-II (2)	Goat (2)
1,727 (3)	O88:H7 (1), novelO:H7 (2)	LT-II (3)	Cow (2), goat (1)
2,521 (1)	O7:H21 (1)	LT-II (1)	Goat (1)
2,539 (2)	O156:H7 (2)	LT-II (2)	Cow (2)
15,796 (2)	O39:H21 (2)	LT-II (2)	Cow (2)
15,797 (1)	O88:H8 (1)	LT-II (1)	Goat (1)
15,798 (1)	H7 (1)	STa (1)	Goat (1)
B2 (7)	9,535 (4)	O53:H45 (4)	LT-II (4)	Cow (4)
9,610 (2)	O103:H45 (2)	LT-II (2)	Cow (2)
13,653 (1)	O2:H45 (1)	LT-II (1)	Cow (1)
Cryptic clade I (7)	2,467 (1)	O2:H25 (1)	STa (1)	Goat (1)
2,715 (2)	O2:H25 (2)	LT-II+STa+stx (2)	Cow (2)
3,057 (2)	O98:H5 (2)	STb (2)	Pig (2)
7,646 (2)	O23:H25 (1), O109:H25 (1)	LT-II+STa (1), LT-II+STa+stx (1)	Goat (1), cow (1)
D (3)	69 (1)	O17:H18 (1)	LT-II (1)	Cow (1)
108 (1)	O129:H15 (1)	STb (1)	Goat (1)
2,944 (1)	O17/O106:H28 (1)	STb (1)	Goat (1)
E (2)	7,701 (1)	O24:H41 (1)	STa (1)	Goat (1)
14,939 (1)	O15:H45 (1)	LT-II (1)	Cow (1)

Serotype diversity was notable across the isolates, with four new O-serotypes identified in each of the livestock types, all within phylogroup B1. Serotype variation was observed in phylogroup B1, including somatic antigens (O-antigens), such as O102, O45 and O88. In contrast, phylogroup B2 was more restricted in serotype diversity, predominantly associated with H25 antigen and O-antigen O53, O103 and O2, and mostly found in cattle (Fig. 1a).

MLST analysis further showed distinct phylogenetic clustering based on STs. ST9535 and ST9610, both associated with phylogroup B2, were predominantly linked to cattle. In contrast, phylogroup B1 encompassed a wider variety of STs and livestock type, suggesting a broader host range (Table 2).

In total, four new O-antigen serotypes were identified, with serotype diversity observed in phylogroup B1. While phylogroup B2 was more limited in both serotypes and host-livestock, phylogroup B1 exhibited significant variation across multiple genetic markers, underscoring the complex epidemiology of ETEC in livestock.

Of the 60 ETEC isolates, 3 had genome sizes exceeding 6 Mb, 2 had more than 600 contigs and 1 exhibited reverse toxin orientation (with subunit B upstream of subunit A). These six isolates were excluded from the core genome analysis to ensure accurate gene-to-gene comparisons. Phylogenetic analysis of the 54 core genomes revealed clustering that was consistent with phylogroup distinctions, and further diversity was observed across livestock type, serotype and MLST (Fig. 1a). These 54 isolates were also used to allocate toxin alleles and adhesin variants (Fig. 1b).

This analysis highlights the substantial genetic diversity among ETEC isolates, particularly in phylogroup and serotype distribution, with important implications for understanding ETEC epidemiology and host adaptation in livestock. Sequencing runs, assembly statistics, metadata and other attributes are available in Table S3.

### Genetic diversity of LT and ST enterotoxins

Among the 60 animal ETEC isolates, 46 (76.7 %) exhibited the LT toxin, distributed as follows: LT-II only (*n*=37), LT-IIa2−01+STa (*n*=4), LT-I only (*n*=1) and one isolate with reverse LT orientation (*n*=1). Three genomes (ERS7856840, ERS7856842 and ERS7856849) were excluded from LT-specific analysis due to truncated *eltA* or *eltB* genes at contig ends, probably from incomplete assembly.

**Fig. 2.**
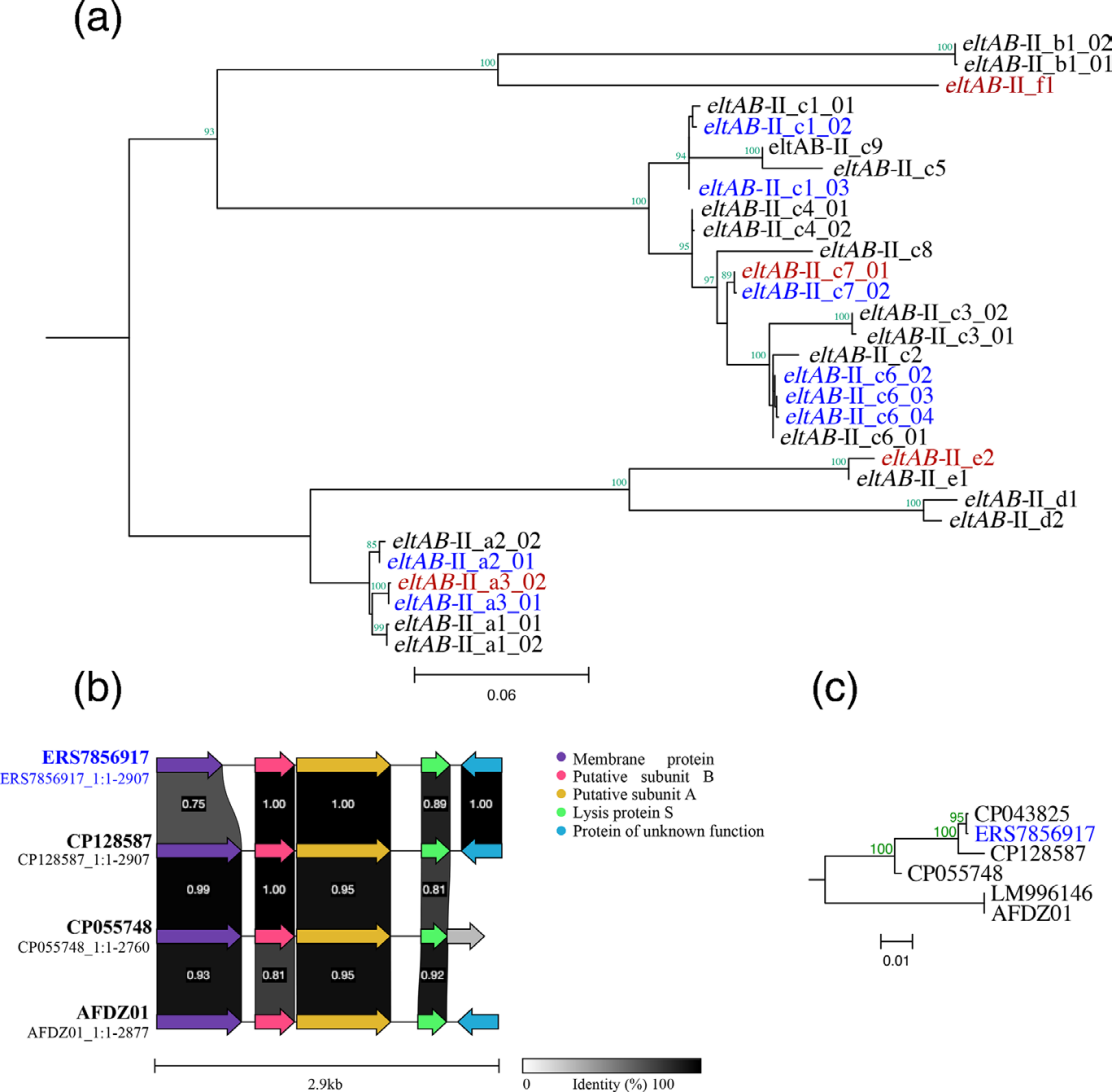
Phylogenetic placements of global LT-II alleles are shown in part (a). Syntenic comparison of the labile toxin with reverse orientation with related genomes from other studies (in black) is shown in part (b) and its phylogenetic relation in part (c). Alleles in blue font represent isolates from this study and in red are from other Kenyan studies.

The 41 LT-II isolates were related to either LT-II-a (*eltAB*-II-a, 17.1%, 7/41) or LT-II-c (*eltAB*-II-c, 82.9%, 34/41). Eight novel LT-II alleles were identified across 24 livestock ETEC isolates: *eltAB*-II-a2-01 (*n*=4), *eltAB*-II-a3-01 (*n*=3), *eltAB*-II-c1-02 (*n*=9), *eltAB*-II-c6-02 (*n*=3), *eltAB*-II-c6-03 (*n*=3), *eltAB*-II-c6-04 (*n*=1) and *eltAB*-II-c7-02 (*n*=1). No *eltAB*-II-b alleles were detected. A total of 30 *eltAB*-II alleles were identified with publicly available nt sequences incorporated (Fig. 2a).

One isolate from a bull in Shinyalu contained a human-specific LT-I allele (*eltAB*-I-15). Another cow isolate exhibited reverse toxin orientation. Its putative B-subunit, 333 bp long, shared 15.2% and 19% aa identity with LT-I-15 (*eltB*-I-15) and LT-II-c9 (*eltB*-II-c9-01), respectively. The A-subunit, 792 bp long, was more distantly related to LT-II (*eltA*-II-c9-01, with 57.7% aa identity) than to LT-I (*eltA*-I-15, with 50.4% aa identity). The B- and A-subunits were flanked by an upstream putative membrane protein gene and a downstream lysis protein S gene, alongside other prophage genes (Fig. 2b). This arrangement has been observed in other *E. coli* genomes globally with three probable alleles (Fig. 2b, c).

Fourteen genomes had ST-only toxin distributed as follows: STa only (*n*=5), STb only (*n*=7) and STa+STb (*n*=2). STa-only positive ETEC isolates were found in goats and sheep, with STa-6-02 (*estA*-6-02) detected in two goats and one sheep and STa-4-02 (*estA*-4-02) in one goat. Another goat isolate carried both STa-4-02 and STa-5-01 (*estA*-4-02 and *estA*-5-01).

STb was present only in pig and goat isolates (Fig. 1b). Caprine-associated STb was STb-2-1 (*estB*-2-01), while swine-associated STb was STb-1-01 (*estB*-1-01) and STb-1-04 (*estB*-1-04). STb-1-04 (*estB*-1-04) in pig ETEC isolates also exhibited concurrently with STa-1-02 (*estA*-1-02).

**Fig. 3.**
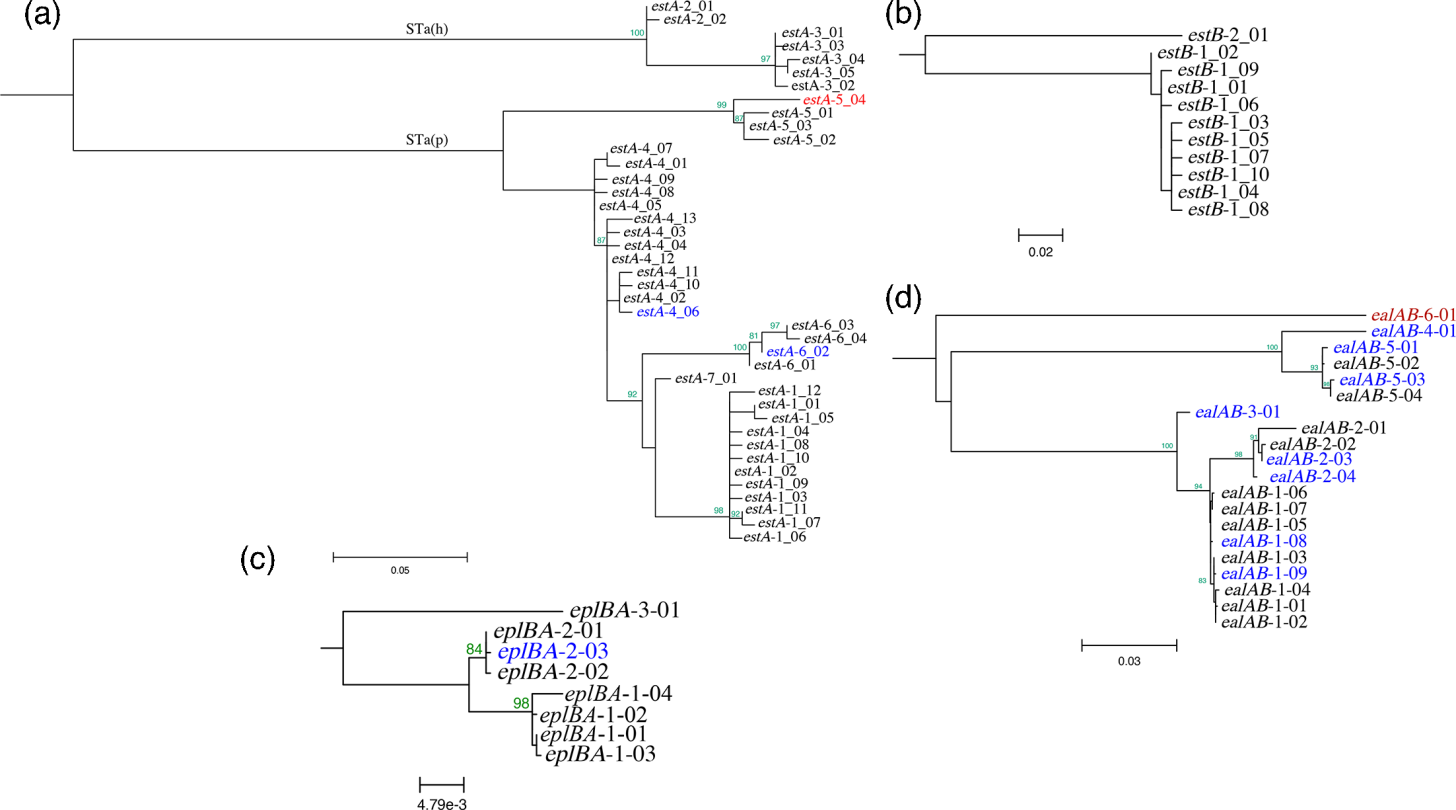
Phylogenetic placements of global STa alleles are shown in part (a), STb in part (b) and Epl and Eal in parts (c) and (d), respectively. Alleles in blue font are from this study, while those in red are from a separate study in Kenya. Those in black font are from other parts of the world or previously described, or in publicly available databases.

Three bovine isolates exhibited LT-II-a2-01+STa-4-02 (*eltAB*-II-a2-01 and *estA*-4-02) toxin combination, while one goat ETEC had LT-II-a2-01+STa-4-06 (*eltAB*-II-a2-01 and *estA*-4-06). These four LT+ST isolates were from cryptic clade I. Overall, by incorporating publicly available nt sequences, STa toxins clustered into 41 *estA* alleles, while STb clustered into 11 *estB* alleles (Fig. 3a, b).

### Pertussis-like toxins in ETEC strains

*E. coli* ArtAB-like (Eal) and *E. coli* PltBA-like (Epl), encoded by the *EalAB* and *EplBA* operons, are heteropentameric holotoxins related to the aerolysin/pertussis toxin heptamer, produced by *Bordetella pertussis*, a mucosal epithelium pathogen that causes whooping cough [18,55]. EalAB and EplBA, first found in LT-II-positive isolates, are typically separated by three phage-related genes – two putative phage holins and a lysozyme. Of the 46 LT-positive genomes, six genomes (ERS7856848, with LT-I and ERS7856917 with the reverse LT order, and four with a typhoid-like toxin) did not exhibit Eal or Epl toxins. Three ST-positive genomes carried these pertussis-like toxins, resulting in a total of 43 livestock ETEC genomes with Eal or Epl toxins.

Epl toxin was present in 20 LT-II-only genomes. However, four genomes (ERS7856835, ERS7856840, ERS7856843 and ERS7856849) were excluded from the Epl-specific analysis due to truncated *eplA* or *eplB* genes, likely due to incomplete assembly with gene ends positioned at contig edges. In the remaining ETEC genomes (*n*=16), *Epl* alleles were distributed as follows: Epl-1-01 (*eplBA-1-01*, *n*=4), Epl-2-01 (*eplBA-2-01*, *n*=9), Epl-2-02 (*eplBA-2-02*, *n*=2) and Epl-2-03 (*eplBA-2-03*, *n*=1). In total, the *eplBA* genes clustered into eight alleles (Fig. 3c), with one novel allele (*eplBA-2–03*) found exclusively in livestock from Western Kenya.

Among the 23 isolates positive for the Eal toxin, associated distribution with ETEC toxins included LT-II-a2+STa-4 (6.7%, 4/60), LT-II only (26.7%, 16/60), STa+STb (1.7%, 1/60) and STa only (3.3%, 2/60). Within these, allele distribution was as follows: Eal-4-01 (*ealAB*-4-01, *n*=6), Eal-5-01 (*ealAB*-5-01, *n*=6), Eal-2–03 (*ealAB*-2-03, *n*=3), Eal-1-09 (*ealAB*-1-09, *n*=2) and one each of Eal-1-05 (*ealAB*-1–05), Eal-1-08 (*ealAB*-1-08), Eal-2-01 (*ealAB*-2-01), Eal-2-02 (*ealAB*-2-02), Eal-3-01 (*ealAB*-3-01) and Eal-5-03 (*ealAB*-5-03). In total, the identified *ealAB* genes clustered into 20 alleles (Fig. 3d), 6 of which were novel alleles specific to livestock from Western Kenya (*ealAB*-1-09, *ealAB*-2-03, *ealAB*-3-01, *ealAB*-4-01, *ealAB*-5-01 and *ealAB*-5-03).

These pertussis-like toxins are worth investigating as potential additional toxins, given their close association with animal ETECs, suggesting a possible role in enhancing the pathogenicity of *E. coli* isolates from livestock. Allele accession numbers for LT-II, STa(p), Eal and Epl are available in Table S4.

### Putative typhoid-like toxin with an A_2_B_5_ potential

**Fig. 4.**
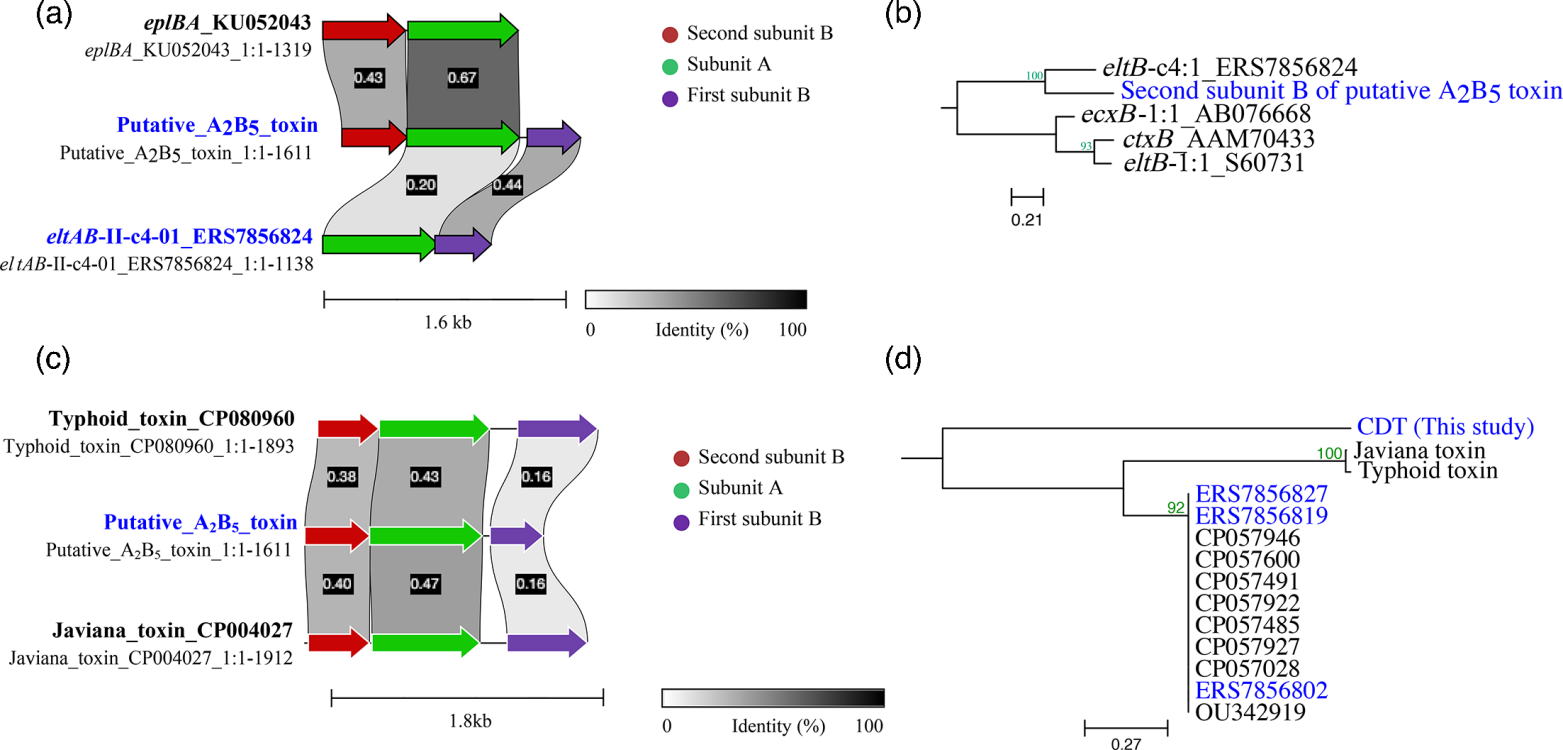
The second B-subunit in the putative A_2_B_2_ toxin is distantly related to LT-II-c, while the first subunit and the A-subunit are related to EplBA than to typhoid toxins. In this figure, sections (a) and (b) show the relatedness of the second B-subunit to selected AB_5_ toxins, while sections (c) and (d) are specific for known A_2_B_5_ toxins. aa synteny is shown on closely related typhoid toxins with decimal identities appended in white font on black background in sections (a) and (c).

In four Epl/Eal-negative, LT-II-positive strains, a putative tripartite toxin with a probable A_2_B_5_ architecture was identified. These strains, isolated from three cows and one pig (MLN), exhibited a toxin comprising a three-gene locus: two B-subunits flanking a catalytic pertussis-like A-subunit. The upstream B-subunit is shared aa sequence synteny with *eplB*, while the downstream B-subunit was syntenic to *eltB* of LT-II-c. To substantiate these findings, a maximum-likelihood phylogenetic tree was constructed, comparing the B-subunit with representative AB_5_ and A_2_B_5_ toxins (Fig. 4a, b). Unlike *eplB*, the putative toxin lacks DVSS repeats downstream of the predicted signal peptide sequence. The A-subunit of the typhoid-like toxin shared 66.4% aa identity with *eplA*, while the downstream B-subunit showed 44.5% identity with *eltB*-II-c4-01 from this study.

Predicted protein modelling of the first B-subunit revealed a pentameric structure, with 52.1% aa identity to a subtilase toxin found in *E. coli* (PDB entry 3DWA). Subunit A showed 66.8% identity to a subtilase toxin (PDB entry 4Z9C), and the downstream B-subunit showed 51.6% identity with LT-II-c pentamer (PDB entry 7PRP). Additionally, eight publicly available genomes with the same A_2_B_5_ operon sequence as the LT-II-positive ETEC isolates in this study were incorporated into the analysis and showed that the operon spans 1,611 bp (Fig. 4a).

aa synteny analysis and maximum-likelihood phylogeny of this A_2_B_5_ toxin showed a closer similarity to typhoid toxins in *Salmonella* spp. compared to CDT in *E. coli* (Fig. 4c, d), emphasizing its distinctive relationship within the A_2_B_5_ toxin family. The putative novel A_2_B_5_-like toxin identified warrants further experimental investigation to elucidate its functional role and contribution to pathogenesis.

*Note: Javiana toxin has 99% identity to TT and is present in other Salmonella spp. serovars*.

### ETEC hybrids

Among the analysed isolates, four (6.6%, 4/60) were identified as ETEC/STEC hybrids, carrying either *eltAB*-II+estA+*stxAB*-2 or *estA+estB+stxAB*-2. All ETEC/STEC hybrids in this study possessed the haemolysin genes *hlyCABD*. Three hybrids, isolated from cattle, carried LT-II-a2-01 (*eltAB*-II-a2-01), STa-4-02 (*estA*-4-02), Stx2-g (*stxAB*-2g) and a subtilase-like toxin [Eal-2-03 (*ealAB-*2-03) or Eal-2-04 (*ealAB-*2-04)] positioned upstream of *eltAB*. Strain ZOL072668 was isolated from a cow slaughtered at Lubao, while the other two strains were sampled from the Funyula livestock market with a 1-month interval. These cattle isolates were associated with *E. coli* cryptic clade 1 and phylogroup A.

The remaining identified hybrid strain was obtained from a pig and carried STb-1-04 (*estB*-1-04), STa-1-02 (*estA*-1-02), Stx2-e (*stxAB*-2e) and a subtilase-like toxin [Eal-1-09 (*ealAB*-1-09)] positioned upstream of *eltAB*. This pig strain also belonged to phylogroup A.

Two ETECs with CDT were identified in this study, with each carrying the *cdtABC* operon flanked by IS21 and IS2 transposases. One isolate exhibited both CDT (*cdtABC*) and STb-1-01 (*estB*-1-01), from an MLN of a male pig slaughtered at Lubao pig slab on 28 June 2018. This isolate was associated with phylogroup A, serogroup O132:H25, ST93 and had three plasmid replicons: IncFIC, IncFIB and IncFIA.

The second ETEC with CDT exhibited both LT-II-c1-01 (*eltAB*-II-c1-01) and a truncated A_2_B_5_-type toxin with the A-subunit truncated at the operon’s first subunit-A positioned at a contig edge but found disrupted in another contig edge. This cow isolate, containing three AB_5_ toxins, was associated with phylogroup B1, serotype O45:H10, ST711 and had one IncFIB plasmid replicon. The *cdtABC* operon in both hybrids shared 99.8 % aa identity with the type I *cdtABC* operon in CP028192.1, an *E. coli* strain isolated from Danish pig tissue, differing only by an M1V substitution in *cdtA*.

### Putative adhesins and associated characteristics

**Fig. 5.**
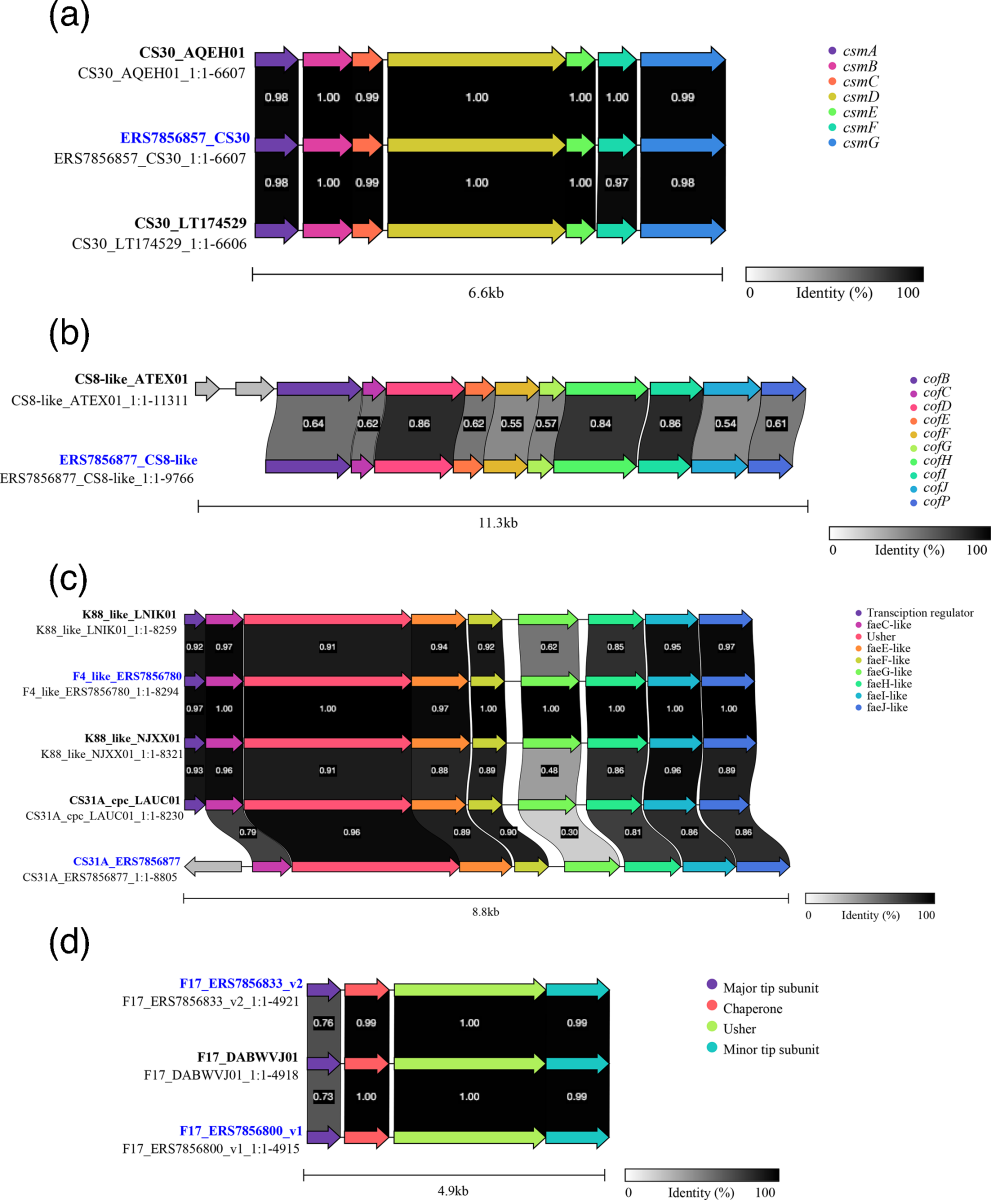
Syntenic gene-to-gene comparison of CFs found in humans; CS30 [part (a)] and CS8 [part (b)], while K88 [part (c)] and F17 [part (d)] adhesins are found in animals. Operons from isolates from this study are shown in blue font. Those in black are previously described or from publicly available databases.

Animal ETEC isolates in this study were notably negative for the classic adhesins F4, F5 and F6. However, CS31A-like adhesins, encoded by *clp*-like operon, were identified in cattle and one goat isolate. Two CS31A-like variants were observed: variant 1, present in ETEC/*stxAB-2g* hybrids from cattle, and variant 2 in a goat ETEC isolate. With intergenic regions excluded, variant 1 showed 84.7% aa identity to *clpC-J* region in entry LAUC01000178, while variant 2 shared 82.8% identity. Two F17-like adhesin variants (designated as alleles 22 and 23) were identified in LT-II-positive animal ETEC strains. F17 : 22 was observed in cattle and showed 94.2% aa identity to F17A:1 (AF022140), while F17 : 23, with 94.6% identity, was found in a goat isolate (Fig. 5c, d).

Adhesins with sequence similarity to, or identical to, known CFs in human ETEC were identified in the animal isolates. Using a blast-based approach, a CS30-like adhesin was identified in an ETEC/STEC hybrid isolate from a pig sampled at Ikolomani. The query sequence, derived from LT174529 [56], showed a large-scale blast score ratio value of 0.9795, indicative of a high syntenic match (Fig. 5a). Similarly, a putative gamma-2 ETEC pilus (*gep*) identical to the one previously described [57] was present in an LT-I-positive isolate from a cow, alongside a putative P-like fimbriae. Additionally, a goat ETEC strain exhibited a truncated type IV pili syntenic to CS8 (Fig. 5b).

**Fig. 6.**
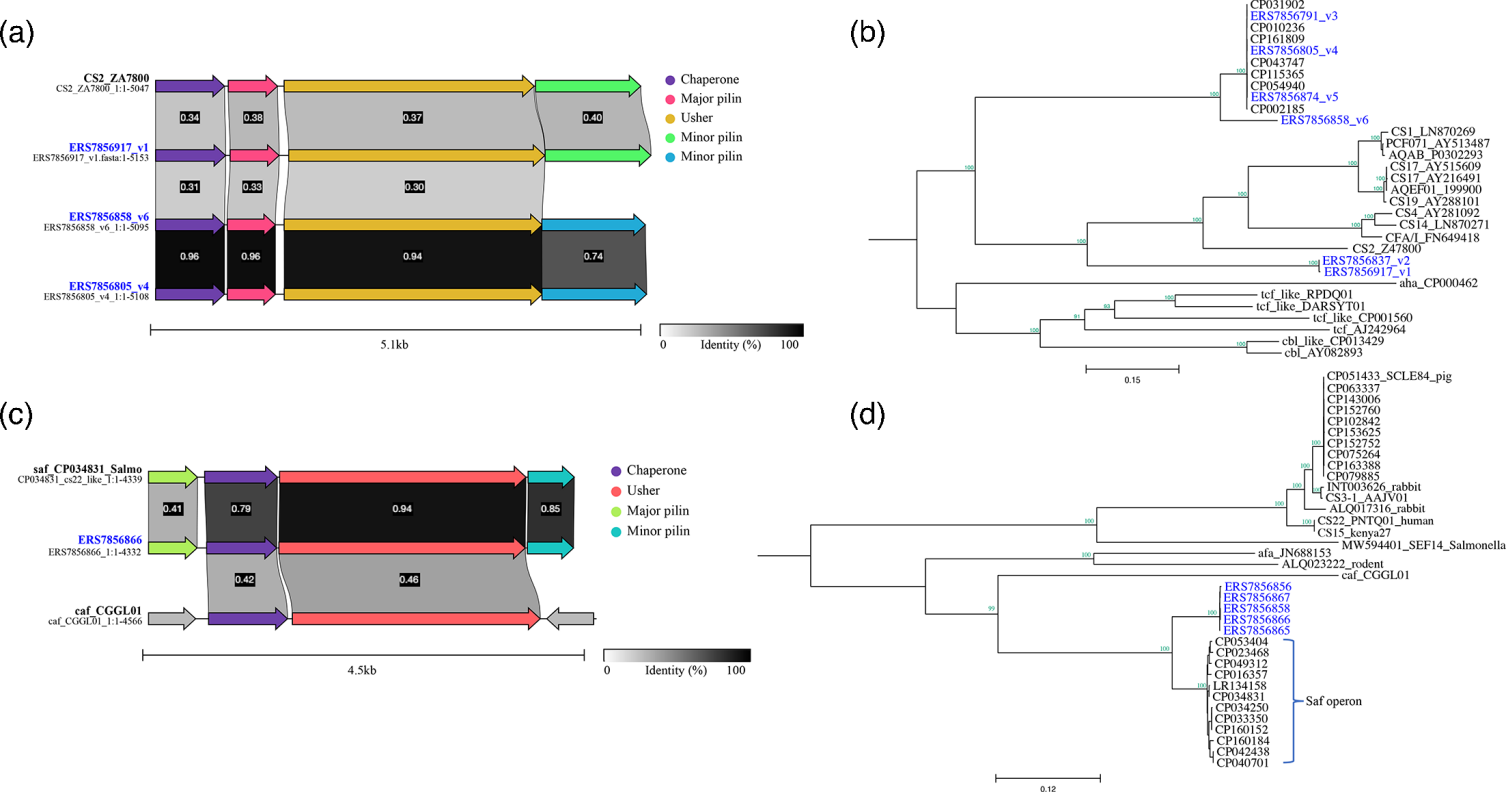
Comparison of class 5-like fimbriae or *α*-like fimbriae in relation to other members is shown in syntenic gene-to-gene comparisons in part (a) and phylogenetic placements in part (b). γ3-like adhesin syntenic gene-to-gene comparison with the related saf operon is shown in part (c), and phylogenetic placement with other members is shown in part (d). Blue font entries are from this study while those in black font are from other regions of the world, previously described or from publicly available databases.

Class 5 fimbriae are part of the *α*-family of chaperone-usher CFs in human ETECs, comprising eight antigenically distinct types: PCFO71, CFA/I, CS1, CS2, CS4, CS14, CS17 and CS19 [58,60]. In this study, adhesins distantly resembling class 5 fimbriae were identified in 58.3% (35/60) of animal ETECs, represented by 6 distinct variants for reference: variant 3 (v3) in 28 isolates, variant 4 (v4) in 4 isolates from all 4 livestock types, variant 5 (v5) in a goat and variant 6 (v6) in 2 pigs. Variant 6 spanned 5,095 bp long, while v3, v4 and v5 each are 5,108 bp. These four variants represent adhesins distantly related to human CFs belonging to class 5 fimbriae. However, two variants, variant 1 (v1) and variant 2 (v2), were closer phylogenetically, to human CFs belonging to this family. These two variants were identified in cow ETEC isolates. Gene-to-gene comparison and phylogenetic placements of these class 5-like fimbriae with known human ETEC CFs and other related class 5 fimbriae family members are shown in Fig. 6(a, b).

The γ3-fimbriae are classified by usher aa synteny and the presence of an extended F_1_-G_1_ loop, a structural feature of their chaperones [61]. Although this group includes a variety of adhesins, operons such as *Salmonella* atypical fimbriae (saf), identified in clinical isolates of *S. enterica*, CS15, CS22 (from human CFs) and CS3-1 (from rabbit EPEC), were the most comparable adhesins for gene-to-gene evaluation with the γ3-like adhesin identified in this study [61,65]. A γ3-like adhesin with five alleles was identified exclusively in five pig ETECs, showing a distant relation to CS15 and CS22. The operon consists of a 495 bp major structural subunit gene, a 738 bp chaperone, a 2,511 bp usher and a putative pilin tip (471 bp). The five alleles varied by one to eight aa substitutions within the major structural gene, particularly between positions 59 and 152. Phylogenetic analysis revealed that the putative γ3-like adhesin in pigs showed distant phylogenetic relation from the human-associated CS15 and CS22 CFs, with its closest relative being the orthologous *saf* operon found in *S. enterica* (Fig. 6c, d).

## Discussion

The three counties included in this study, Busia, Bungoma and Kakamega, were chosen to represent the broader Lake Victoria basin ecosystem. This region, characterized by high rural human and livestock population densities in East Africa, operates under a mixed smallholder livestock production system, making it a critical site for zoonotic disease surveillance. The suitability of this area as a One Health integrated surveillance site for zoonotic diseases was previously emphasized [37]. Slaughterhouses and livestock markets served as convenient and converging sampling points for ruminants (cattle, goats and sheep) in this study’s context. However, due to the absence of formal slaughterhouses or slabs in Bungoma County, pig samples were collected exclusively from Busia and Kakamega. Insufficient economic motivation to establish such facilities was likely caused by the relatively low demand for pork in Bungoma. Therefore, unlike other animals, all pig samples were exclusively collected from slaughterhouses/slabs as part of the overall sampling strategy adopted in the parent study [37].

MLNs were selected for sampling due to their proximity to gut *Enterobacteriaceae*, which can infiltrate these nodes through translocation or other dynamics based on the host immune condition. Via the lymphatic system, MLN-sequestered bacteria can reach peripheral lymph nodes, e.g. subiliac, popliteal and superficial cervical lymph nodes, potentially contaminating meat and meat products, post-slaughter [66,67]. Therefore, MLNs are valuable sample types in the isolation of *E. coli* and other gut-originating *Enterobacteriaceae*.

Discrepancies between PCR and WGS in pathotype identification were observed. Differences can occur if the two are not done concurrently. In another study, WGS and PCR agreement stood at 83% (29/35) [68] while agreement in this study was lower (61.2%, 60/98). Prophage loss in a longer initial PCR-pathotyping passage to WGS passage interval could be an unevaluated reason for this, especially in chromosomally encoded *eltAB*-II genes and plasmid loss in plasmid-located *eltAB*-I and/or heat-stable (ST) genes. Other reasons could be that primer sequences bound to homologous sites elsewhere in the genome or plasmid-borne toxin genes were missed by the Illumina (short-read) technology during assembly.

Among the ETEC isolates identified via WGS, 71.7% (46/60) carried the LT toxin, with the majority classified under the LT-II-c clade. This clade encompasses nine *eltAB*-II alleles (LT-II-c1-9), initially identified in dead ostriches with diarrhoea [9,10]. Since their discovery, LT-II-a, LT-II-b and LT-II-c toxins have been isolated from various sources, including humans, raw beef, calves, buffalo, sheep, cows and foodstuffs including mayonnaise [69]. In this study, two unique *eltAB*-II-a alleles were designated as *eltAB*-II-a2-01 and *eltAB*-II-II-a3-01, in cattle, goats and sheep. Out of the five *eltAB*-II-a2-01 alleles, four were from cattle and one from a goat, while *eltAB*-II-a3-01 was present in sheep and goats (two goats and one sheep). Allele *eltAB*-II-a3-02 was found from a previous study in Nairobi [70]. No alleles similar to LT-II-b were identified in this study. Additionally, new alleles, *eltAB*-II-c1-02, *eltAB*-II-c6-02, *eltAB*-II-c6-03 and *eltAB*-II-c6-07, as well as *eltAB*-II-c7-02, which was found in a goat, were part of this study’s collection. Allele *eltAB*-II-c7-01, previously identified in the red forest duiker (*Cephalophus natalensis*) from South Africa, shares genetic similarities with the domestic goat. This is attributable to their close evolutionary relationship within the *Artiodactyla* order and the *Bovidae* family. This could suggest a potential commonality in the enterocyte receptor sites between these two animals, which is significant for understanding the genetic epidemiology of ETEC infections across different livestock types. Cumulatively, with additional variants obtained from publicly available databases and/or genomes, there are 30 *eltAB*-II alleles. These findings contribute to the expanding genetic diversity of phage-encoded enterotoxins, which are often overlooked. Experimental evidence supports the role of LT-II-b and LT-II-a toxins in causing diarrhoea, particularly LT-II-b and LT-II-a [11]. It is worth noting that most of the LT-II-carrying ETEC isolates worldwide were collected in the 1980s [69], indicating a possibility of identifying additional alleles in isolates collected in 2017–2019. Although LT-II-c has been associated with diarrhoea in a small number of isolates [69], most of the animals sampled in this study exhibited normal faecal consistency, and body temperature was not identified as a risk factor for ETEC infection. It is important to consider that the absence of clinical symptoms does not necessarily imply a lack of biological significance in terms of differences observed. Consistent with previous findings by Jobling *et al*., LT-II-c toxins were the most abundant, and all previously identified *eltAB*-II-c alleles were present in our collection. Limited experimental data exist on the labile toxin with reverse operon orientation ability to cause diarrhoea. These particular toxins have been found in sources varying from cattle faeces, farm environment and patients with a toxin scout on these genomes showing association with the stx1 gene. Additionally, isolates carrying this labile toxin with reverse orientation do not have pertussis-like toxins and potentially harbour adhesin distantly resembling class 5 fimbriae, justifying the need to investigate the interplay between these VFs and their impact on disease in humans and animals.

STa enterotoxins are known to be causative agents of secretory diarrhoea in both humans and animals and have garnered significant attention due to their relevance to public health [14]. STa toxins can be further classified into STa(p) and STa(h) alleles, which are phylogenetically distinct. The current allele categorization cumulatively includes 15 *estA* unique alleles, with debatably human-specific *estA*-2 and *estA*-3 clades, while *estA*-1, *estA*-4, *estA*-5 and *estA*-6 clades can be found in humans and animals [7]. To support this, *estA*-2 and *estA*-3 were not detected in our animal ETEC collection. By incorporating publicly available nt sequences from GenBank and data from two separate Kenyan studies, the total number of *estA* alleles expanded to 41, with one new allele identified, unique to Western Kenya livestock: *estA*-4-06, while *estA*-5-04 was identified from a human in the Nairobi study. The unique allele *estA*-4-06 was isolated from a goat. STb enterotoxins are classified into two main *estB*-1 and *estB*-2 clades. However, with the inclusion of publicly available sequences from GenBank, the global number of *estB* unique alleles expanded to 11 (*estB*-1 to 10 and *estB*-2). Interestingly, the study identified animal-specific *estB*-2-01 allele that was found exclusively in goats. These findings contribute to our understanding of the genetic diversity of STa and STb toxins, highlighting the presence of previously unreported alleles and their distribution across different livestock types.

EplBA and EalAB toxins were first described in 2016 and share the ability to bind to sialylated galactose residues, similar to *pltB* from *Salmonella* spp. The EplBA toxin is structurally similar to PltBA from *S. enterica* serovar Typhi, while EalAB is closely related to ArtAB encoded by *S. enterica* serovar Typhimurium. These toxins, including PltBA, ArtAB, EplBA and EalAB, are encoded by prophages [18]. In this study, additional alleles were identified, totalling 8 in *eplBA* and 20 in *ealAB*, expanding the repertoire of pertussis-like/subtilase-like toxins in *E. coli*.

The identification of Stx2g was first reported in bovine *E. coli* isolates [71]. Three cattle cryptic clade I-ETEC/STEC hybrids harboured *eltAB*-II-a2-01, *estA*-4-02, *stxAB*-2g and *ealAB*-2-02/03 resulting in three AB_5_ toxins. The presence of *stxAB*-2g and *estA*-4-02 has been reported before in clinical isolates from Sweden [72]. However, these clinical isolates did not harbour LT-II, Eal toxins, in addition to differing phylogroups, serotypes and STs found in our animal ETEC collection. Oedema disease in pigs is associated with Stx2e, and its presence is detected occasionally in humans who have uncomplicated diarrhoea or asymptomatic carriers [73]. Stx2e has been detected in patients with diarrhoea from Maasailand [74]. One phylogroup-A ETEC/STEC hybrid with *estA*-1-02, *estB*-1-04, *stxAB*-2e and *ealAB*-1-05 was from a pig’s MLN. Interestingly, one serotype O100:H20 isolate described to harbour *stx*-2e and *estA*-1 (without *estB*) recovered from human infection from a study in Brazil shares the serotype and some allele similarities with pig ETEC/STEC hybrid isolate [75].

Of particular interest, a toxin related to EplBA, rather than PltBA, was identified in LT-II strains in this study. This toxin however contains an additional LT-II-like subunit B, suggesting the potential formation of an A_2_B_5_ toxin structure. It was also found in ETEC genomes from two sheep, five cattle and one isolated from an underground reservoir from a study in the UK [76]. This putative tripartite toxin is also related to the TT and distantly related to CDT, suggesting its potential classification within the A_2_B_5_ family of toxins. While bioinformatic-based predictions have their limitations, it is worth noting that the TT was initially predicted using bioinformatic tools and subsequently validated through laboratory-based experiments [19].

ETEC/CDT hybrids were identified in our strain collection. One Clermont type B1, serotype O45:H10, cow isolate harboured *eltAB*-II-c1-01, *cdtABC*-I and new A_2_B_5_ toxin. The other ETEC/CDT hybrid, Clermont type-A, serotype O132:H25 strain was from a pig and carried *estB*-1-01 and *cdtABC*-I genes. To the best of our knowledge, this is the first report of ETEC/CDT hybrids in Africa at large and Kenya.

Pigs are known hosts for ETEC toxins and adhesins, including K99, K88, F17, F18 and F41 adhesins, which have been linked to oedema disease, neonatal diarrhoea and post-weaning diarrhoea. However, STb and/or LT-II-positive isolates are not often associated with their respective adhesins in other animals. For example, post-weaning pigs in Hungary and Korea have been reported to carry toxin/adhesin combinations such as STa+STb+F18 and STb+STa+StxAB-2+F18 [77,78]. In Spain, sheep were found to carry STa+F41 or STa+F41+K99 combinations [32]. Neonatal diarrhoea in calves is predominantly associated with STa+F5 and/or F41, although there has been a decline in reported F5 prevalence over the years [79]. In this study, we did not find F5 adhesins in ETECs from cattle, which had an average age of 2.98 years. It is possible that not targeting sampling towards diarrhoeic adult cattle may have limited the chances of detecting F5, considering the limited information on F5 vaccination initiatives in the region. Human CFs CS13 and CS23 are related to adhesins F4 and CS31 found in animals [80]. In most CS23-ETEC genomes, two putative genes downstream of *aalH* are hypothetically part of the operon, spanning 777 and 912 bp, respectively, and adjacent to an autotransporter, *tleA* [81]. There is less codon synteny (~10%) on the first gene downstream of *aalH* compared to the CS31A-like gene in the same position. However, the second 879 bp gene shares ~83% aa identity with the similar 912 bp gene in the CS23 extended operon. The class 2-like serine protease autotransporters from the two CS31A variants in this study share ~50% and 52% aa synteny with *tleA* and *tsh*, respectively.

In this study, we focused on animal adhesins that are identical or closely related to CFs found in human ETECs. Two such adhesins were identified: a CS30 adhesin from a pig and CS8 from a goat. These findings suggest that animals may serve as reservoirs or sources of spill-over for these strains, considering that they also carried STa.

Although these livestock ETECs cumulatively harboured approximately ten P-pili or Type-1 like fimbriae, the presence of CS31A-like, F17-like, class 5 fimbriae-like and γ-like adhesins in cattle and goats is of interest and warrants their inclusion in current screening methods to investigate their occurrence and association with disease globally.

New *elt*AB-II and *est*A alleles have been identified in this study, cumulatively adding up to 30 *eltAB*-II alleles, 41 *estA* alleles and 11 *estB* alleles. Additionally, 20 *ealAB* and 8 *eplBA* alleles are also reported herein emphasizing the growing diversity of pertussis-like toxins in *E. coli*.

Without matching ST, phylogroup, serotype, *ealAB*, *eltAB*-II or *estB* combinations, ETEC/STEC hybrids (with *stxAB*-2 and *estA*) that were identified in this study have been reported in human infections [72]. The coexistence of these toxins in ETEC/STEC hybrid strains challenges our understanding of their pathogenic potential in animals and underscores the importance of studying their virulence mechanisms in greater detail.

Another interesting finding from this study is the putative toxin with an A_2_B_5_ potential. Its role in disease, ADP-ribosyltransferase activity, crystal structure, toxin delivery mechanisms, vaccine potential and immunotoxin properties remains speculative. These aspects warrant further investigation through experimental studies to unravel the functional characteristics and potential implications of this novel toxin. A major limitation of this study was the lack of experimental validation of the A_2_B_5_-like toxin, the pertussis-like toxins and adhesins identified, leaving their roles in virulence unconfirmed. This gap underscores the need for future studies involving functional assays and structural analyses. Without such investigations, the presence of these putative toxins may simply reflect the classical genome plasticity of *E. coli*, rather than definitive markers of pathogenicity. Advancing our understanding of the putative A_2_B_5_-like-type toxin will require multidisciplinary approaches and collaborative efforts. It is essential to determine its pathogenic potential in WT strains, elucidate its structural features, assess its suitability as a vaccine candidate and explore its immunotoxin properties. These investigations will contribute to a more comprehensive global understanding of the ART family of toxins.

Each new finding snowballs knowledge surrounding ETECs and their associated adhesins/CFs. By unravelling the intricate interactions between these pathogens and their hosts, we move closer to developing effective adhesin or toxin-based vaccines. The study highlights an expanding toxin repertoire and underscores the global importance of monitoring such pathogens.

Overall, while this study offers valuable insights into ETEC toxin and adhesin diversity, the lack of experimental evaluation remains a key limitation. Confirmatory studies are needed to rule out *E. coli* genome plasticity as the sole explanation and to clarify the potential for zoonotic transmission and animal reservoirs in ETEC epidemiology.

## Conclusion

This study provides important insights into the genetic diversity of ETEC strains in Western Kenyan livestock and the broader Lake Victoria Crescent ecosystem of eastern Africa. New LT-II-a and STa alleles, as well as ETEC/STEC hybrids and a putative toxin with A_2_B_5_ potential, were identified. While these findings open avenues for further research and collaboration in adhesin-based vaccine development and allele surveillance, they also underscore a critical challenge: as genomic diversity among ETEC strains becomes increasingly apparent, developing targeted and effective adhesin-based vaccine interventions grows more difficult. Nevertheless, sustained surveillance and research remain vital to combating ETEC-related diseases and safeguarding public health on a global scale.

## Supplementary material

10.1099/mgen.0.001515 Uncited Supplementary Material 1.

## Abbreviations

AB_5_: A-subunit and pentameric B-subunit structure
ART: ADP-ribosylating toxin
CDT: cytolethal distending toxin
CF: colonization factor
cGMP: cyclic GMP
CS: colonization surface antigen
CT: cholera toxin
DVSS: aspartate-valine-serine-serine
Eal: *Escherichia coli* ArtAB-like toxin
EHEC: enterohaemorrhagic *Escherichia coli*
EPEC: enteropathogenic *Escherichia coli*
Epl: *Escherichia coli* PltBA-like toxin
ETEC: enterotoxigenic *Escherichia coli*
LT: heat-labile toxin
LT-I: heat-labile toxin type I
LT-II: heat-labile toxin type II
MLN: mesenteric lymph node
MLST: multilocus sequence typing
O-antigen: somatic antigen
PltBA: pertussis-like toxin subunit B and A
ST: sequence type
STa: heat-stable toxin type A
STb: heat-stable toxin type B
STEC: Shiga toxin-producing *Escherichia coli*
Stx: Shiga toxin
TT: typhoid toxin
VF: virulence factor
WGS: whole-genome sequencing
